# Supplementary material for: Maresin 1 Exerts a Tissue-Specific Regulation of Adipo-Hepato-Myokines in Diet-Induced Obese Mice and Modulates Adipokine Expression in Cultured Human Adipocytes in Basal and Inflammatory Conditions
Source: Biomolecules. 2023 May 31;13(6):919. doi: 10.3390/biom13060919 (PMC10295868; doi:10.3390/biom13060919)
Supplement: Supplementary file 1 [file biomolecules-13-00919-s001.zip › biomolecules-2149051-supplementary.pdf]

Supplementary Materials:

Table S1. Effects of MaR1 on body composition and serum biochemical measurements in DIO mice.

Parameter	Control	DIO	DIO + MaR1
Body weight (g)	26.38 ± 0.76	45.25 ± 0.85 ***	44.30 ± 1.09 ***
Fat mass (%)	16.68 ± 1.49	38.27 ± 0.97 ***	37.33 ± 0.62 ***
Glucose (mmol/L)	6.93 ± 0.45	11.11 ± 0.66 ***	9.14 ± 0.58 $^{*,\#}$
Cholesterol (mg/dL)	157.20 ± 15.48	204.6 ± 10.19 *	210.6 ± 19.70 *
Triglycerides (mg/dL)	123.0 ± 29.35	123.8 ± 18.43	114.8 ± 18.94
ALT (U/L)	92.96 ± 11.51	350.7 ± 38.37 ***	218.1 ±37.34$^{*,\#}$

Data are mean ± SEM (n=7-8). MaR1 (50 μg/kg; 10 days). ALT, alanine aminotransferase; *p < 0.05, ***p < 0.001 vs. Control; #p < 0.05 vs. DIO group.

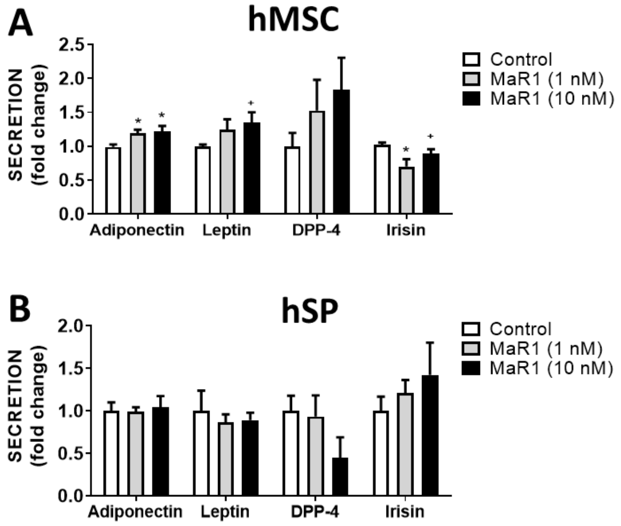

Figure S1. Cumulative secretion (24h) of Adiponectin, Leptin, Dipeptidyl peptidase-4 (DPP4), and Irisin in hMSC-derived and hSP-derived human adipocytes treated with MaR1 (1 and 10 nM) for 24 h. Data from secretion were normalized to their respective controls (n=3-10 from different sets of experiments). Data were analyzed by Student t-test or U-Mann-Whitney and expressed as mean±SEM. *p<0.05, $^{+}$0.05>p<0.1vs. control vehicle-treated cells.

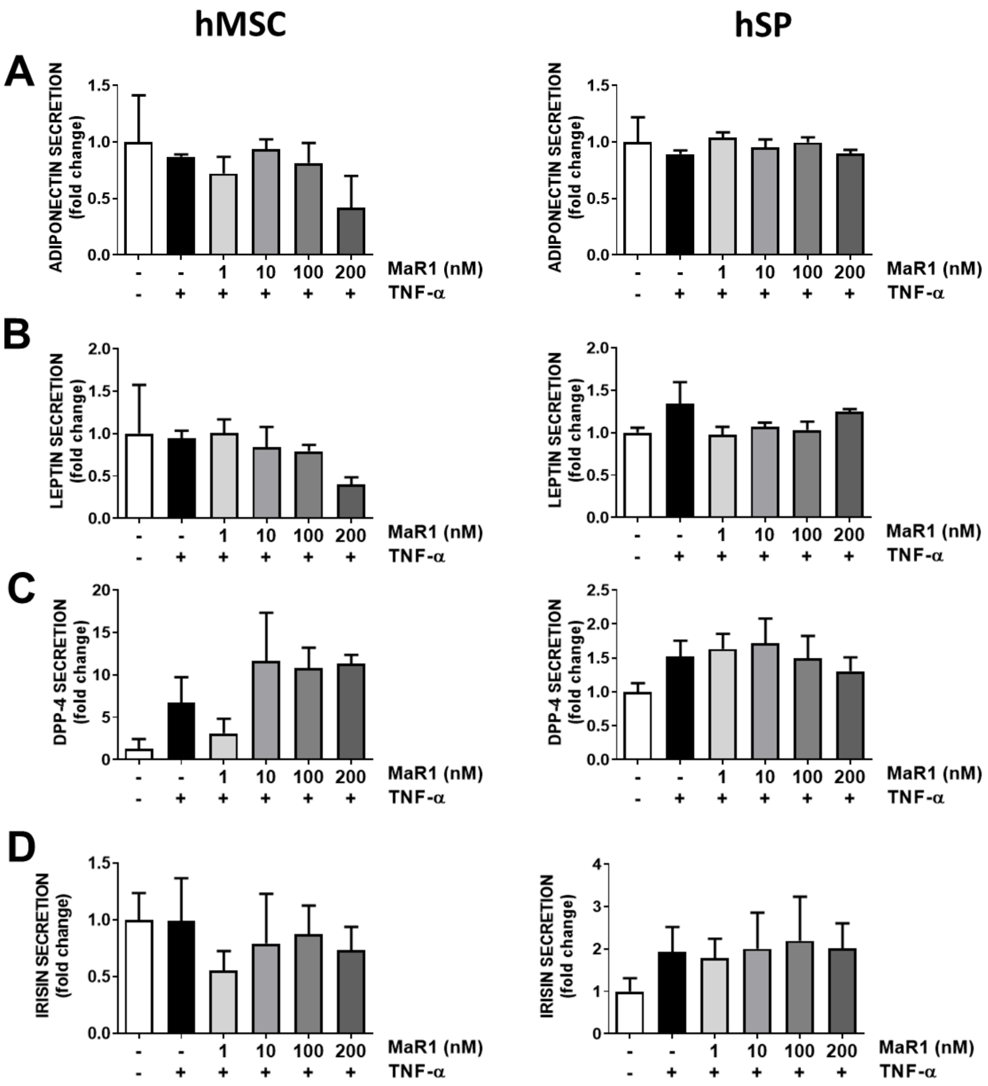

Figure S2. Cumulative secretion (24h) of Adiponectin (A), Leptin (B), Dipeptidyl peptidase-4 ((DPP4, C), and Irisin (D) in hMSC-derived (left panels) and hSP-derived human adipocytes (right panels) treated with TNF-α in the absence or presence of MaR1 (1-200 nM). Data from secretion were normalized to their respective controls (n=3-7 from different sets of experiments). Data were analyzed by One-way ANOVA and expressed as mean±SEM.
